# Modulation of the Gut Microbiota by Shen-Yan-Fang-Shuai Formula Improves Obesity Induced by High-Fat Diets

**DOI:** 10.3389/fmicb.2020.564376

**Published:** 2020-12-21

**Authors:** Zhen Wang, Junfeng Lu, Jingwei Zhou, Weiwei Sun, Yang Qiu, Weihong Chen, Yabin Gao, Ruibing Yang, Sinan Ai, Zhongjie Liu, Yingbo Guo, Wei Jing Liu, Yaoxian Wang, Liang Peng

**Affiliations:** ^1^Department of Nephrology, Dongzhimen Hospital, Beijing University of Chinese Medicine, Key Laboratory of Chinese Internal Medicine of Ministry of Education and Beijing, Renal Research Institution of Beijing University of Chinese Medicine, Beijing, China; ^2^Beijing Key Laboratory for Immune-Mediated Inflammatory Diseases, Institute of Clinical Medical Sciences, China-Japan Friendship Hospital, Beijing, China; ^3^Key Laboratory of Animal Genetics, Breeding and Reproduction of Shaanxi Province, College of Animal Science and Technology, Northwest A&F University, Yangling, China; ^4^Department of Endocrinology, Shengjing Hospital of China Medical University, Shenyang, China

**Keywords:** gut microbiota, obesity, prebiotic, Shen-Yan-Fang-Shuai formula, fecal microbiota transfer

## Abstract

Obesity and related metabolic disorders are associated with intestinal microbiota dysbiosis, disrupted intestinal barrier and chronic inflammation. Shen-Yan-Fang-Shuai formula (SYFSF) is a traditional Chinese herbal formula composed of Astragali Radix, Radix Angelicae Sinensis, Rheum Officinale Baill, and four other herbs. In this study, we identified that SYFSF treatment prevented weight gain, low-grade inflammation and insulin resistance in high-fat diet (HFD)-fed mice. SYFSF also substantially improved gut barrier function, reduced metabolic endotoxemia, as well as systemic inflammation. Sequencing of 16S rRNA genes obtained from fecal samples demonstrated that SYFSF attenuated HFD-induced gut dysbiosis, seen an decreased Firmicutes to Bacteroidetes ratios. Microbial richness and diversity were also higher in the SYFSF-treated HFD group. Furthermore, similar therapeutic effects and changes in gut microbiota profile caused by SYFSF could be replicated by fecal microbiota transfer (FMT). Taken together, our study highlights the efficacy of SYFSF in preventing obesity and related metabolic disorders. Its therapeutic effect is associated with the modulation of gut microbiota, as a prebiotic.

## Introduction

Obesity and related metabolic disorders have become a major global health issue (Morgen and Sorensen, 2014; Arroyo-Johnson and Mincey, 2016; Chooi et al., 2019). There is a wealth of data suggesting that excess body weight and obesity lead to numerous metabolic complications of chronic inflammatory etiology, such as type 2 diabetes, cardiovascular diseases, and non-alcoholic fatty liver disease (NAFLD) (Fan et al., 2017; Verma and Hussain, 2017; Koliaki et al., 2019). In this context, it is imperative to discover effective medical therapies to combat the current obesity epidemic. Traditional Chinese medicine (TCM) is widely used for obesity, diabetes and other metabolic diseases in China. The previous study showed that Shen-Yan-Fang-Shuai formula (SYFSF), composing of Astragali radix, Radix angelicae sinensis, Rheum officinale Baill and four other herbs reduced levels of blood glucose, triglyceride, cholesterol and exhibited anti-inflammatory effects in diabetic mice (Lv et al., 2017). However, it is unknown whether SYFSF is effective for obesity-related disorders.

Recently, there is a growing focus on gut microbiota for the treatment of obesity and its associated metabolic disorders (Chen and Devaraj, 2018; Wang et al., 2018). Evidence from animal and human models strongly supports a relationship between gut microbiome and the development of obesity. For instance, obese ob/ob mice display a decreased number of Bacteroidetes with a corresponding increase in Firmicutes compared to lean mice from the same litter (Ley et al., 2005). Consistent with the results of animal studies, human studies have found that obese individuals have a significantly greater ratio of Firmicutes-to-Bacteroidetes ratio (F/B) than their lean counterparts, although these findings are still a subject of debate (Million et al., 2013). Interestingly, transplantation of gut microbiota isolated from high-fat diet (HFD)-induced obese donor mice into germ-free recipient mice resulted in significant weight gain and metabolic syndrome, which suggesting that changes in gut microbiota occur before the development of obesity (Turnbaugh et al., 2008). Conversely, obesity further worsens the abnormalities of gut microbiota, forming a vicious circle. Furthermore, gut dysbiosis due to obesity suppresses the expression of tight junction proteins, leading to increased intestinal permeability and the translocation of Gram-negative bacteria-derived lipopolysaccharide (LPS) into the blood (Cani et al., 2009). High levels of circulating LPS stimulates Toll-like receptor 4 (TLR4) signaling in various cells (Shi et al., 2006) and leads to metabolic inflammation and insulin resistance in obese mice (Saito et al., 2007). In particular, gut microbiota may provide LPS and other potential harmful molecules to the liver through the gut-liver axis, contributing to establish a chronic low-grade inflammation state and, promoting the NAFLD progression and metabolic syndrome. Thus, modulation of gut dysbiosis by drug or nutritional intervention is a crucial and potential therapeutic target for obesity and its related metabolic disorders (Anhe et al., 2018). Prebiotics are non-digestible food ingredients, which reduce body weight and exert anti-inflammatory effects mainly by enhancing the growth of specific beneficial bacteria found in the gut. Prebiotics not only alter the intestinal microbiota but also improve intestinal permeability and decrease blood endotoxemia caused by LPS. Prebiotics may, therefore, protect against obesity-induced inflammation in animals and clinical study (Genta et al., 2009; Chang et al., 2015; Edrisi et al., 2018; Cerdo et al., 2019; Zeng et al., 2020).

This study aims to investigate whether SYFSY treatment could modulate the composition of intestinal microbiota and thereby alleviate HFD-induced obesity in mice. We also discuss the potential link between the regulation of the intestinal microbiota and the metabolic benefits of SYFSY treatment, which may contribute to an understanding of the potential mechanisms of SYFSY in the prevention and/or treatment of metabolic diseases.

## Materials and Methods

### Herbal Constituents and Extraction

Shen-Yan-Fang-Shuai formula was extracted from seven herbs, namely Astragali Radix (Huang Qi), Rheum Officinale Baill (Da Huang), Radix Angelicae Sinensis (Dang Gui), Sargassum (Hai Zao), Carapax Trionycis (Bie Jia), Concha Ostreae (Mu Li), and Radix Rehmanniae Preparata (Shu Di). The herbs were boiled to obtain a decoction and the final concentration was extracted into 1 g/ml. The herbs were bought from Tongrentang Company, a reputable pharmaceutical company in China known for its rigorous quality-control. Quality control and the final extraction were performed according to established guidelines in the Pharmacopeia of The People’s Republic of China, 2010. The Determination of the related substances in SYFSF by ultra-high-performance liquid chromatography-quadrupole time-of-flight mass spectrometry (UHPLC-Q-TOF) was showed in Supplementary Materials and Methods.

### Animals and Experimental Design

**FIGURE 1:**
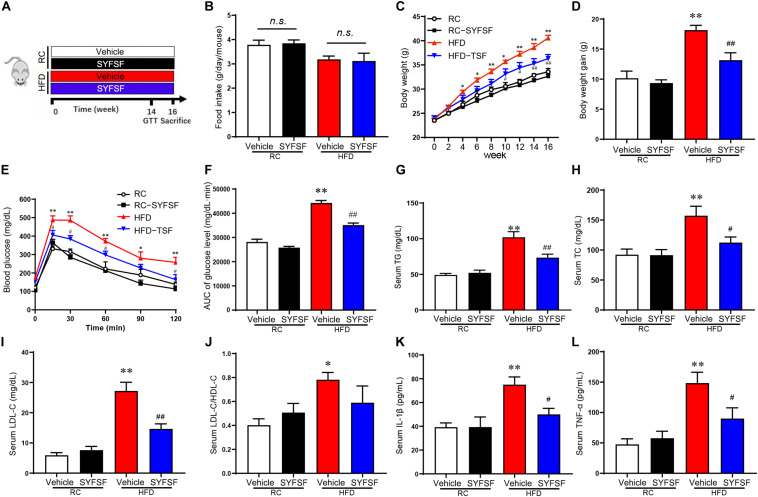
SYFSF prevents weight gain, insulin resistance and low-grade inflammation in HFD-fed mice. **(A)** A schematic diagram for the experimental timeline. **(B)** Food intake. **(C)** Body weight. **(D)** Body weight gain. **(E)** GTT results. **(F)** Area under the curve. **(G–L)** Serum TG, TC, LDL-C, LDL-C/HDL-C, IL-1β, and TNF-α. ^∗∗^*P* < 0.01, ^∗^*P* < 0.05 vs. RC; ^##^*P* < 0.01, ^#^*P* < 0.05 vs. HFD. n.s, not significant. *n* = 6 per group.

Animal study procedures were approved by the Animal Studies Committee of China-Japan Friendship Hospital (Approval No. 20180211). 24 male C57BL/6J mice aged 6 weeks (Beijing Huafukang Bioscience Company, Beijing, China) were bred in a controlled environment and subjected to a 12-h light-dark cycle with free access to food and water. Mice were adapted to the laboratory environment for 2 weeks before conducting the experiment on a regular chow diet (RC, 10% calories from fat, 20% calories from protein, 70% calories from carbohydrate; Beijing Huafukang Bioscience Co., Ltd., H10010). At the age of 8 weeks, the mice were randomly divided into four groups (*n* = 6) and fed with RC or HFD (60% calories from fat, 20% calories from protein, 20% calories from carbohydrate; Beijing Huafukang Bioscience Co., Ltd., H10060). Treatment was carried out concomitantly with HFD feed and consisted of daily intragastric gavage SYFSF (11.4 g/kg/d) or the vehicle (0.5% CMC-Na) for 16 weeks. This dosage of SYFSF was determined based on a previous study demonstrating the protective effect of SYFSF on diabetic kidney disease (Lv et al., 2017). A schematic diagram for the experimental timeline is shown in Figure 1A. Body weight and food intake were measured weekly. At week 16, mice were anesthetized in chambers saturated with isoflurane after a 12-h starvation and sacrificed by cardiac puncture. Serum samples were obtained and centrifugated at 3000 rpm at 4°C for 10 min. Organs and tissues were collected, weighed and frozen at −80°C.

### Glucose Tolerance Test

Glucose tolerance test (GTT) was performed according to standard protocol (Wu et al., 2011). Mice were fasted overnight (16 h), and subsequently given an intraperitoneal glucose injection (2 g/kg body weight). Blood glucose levels were then measured at 0, 15, 30, 60, 90, and 120 min after the injection using a Contour Glucose Meter and Contour Glucose Strips (Bayer, Berlin, Germany).

### Serologic Analysis and Serum Endotoxin Detection

The 3100-type automatic biochemical analyzer (Hitachi, Tokyo, Japan) were used to measure the level of alanine aminotransferase (ALT), aspartate aminotransferase (AST), low-density lipoprotein-cholesterol (LDL-C), high-density lipoprotein-cholesterol (HDL-C), triglycerides (TG) and total cholesterol (TC) levels in mouse serum samples. Serum levels of tumor necrosis factor-α (TNF-α) and interleukin-1β (IL-1β) were measured using ELISA kits (R&D Systems, Minneapolis, MN, United States). Serum endotoxin was extracted and heat-inactivated at 70°C for 15 min before measuring with a Limulus amebocyte lysate (LAL) kit (Cambrex BioScience, Walkersville, MD, United States). This is in compliance with standard protocol for endotoxin testing (Wu et al., 2019).

### Histology and TG Analyses of the Liver

Hematoxylin and eosin (H&E) staining and Oil Red O staining were used to visualize histological morphology and lipid deposition in liver tissue. Before each staining, liver tissue was fixed in 4% paraformaldehyde solution for 24 h, and embedded in paraffin. For H&E staining, 4 μm transverse sections were cut and processed according to standard protocol (Cardiff et al., 2014). For Oil Red O staining, 6 μm sections were cut and stained with Oil Red O (Sigma-Aldrich, St. Louis, MO, United States) for 20 min. All stained sections were examined using light microscopy (×200 magnification). TG concentrations in the livers (30 to 50 mg in weight) were extracted and determined using a commercial kit (Nanjing Jiancheng Bioengineering Institute, Nanjing, China).

### Quantitative Polymerase Chain Reaction (qPCR)

Total RNA extraction, reverse transcription, and quantitative polymerase chain reaction (qPCR) were performed according to standard protocol (Wu et al., 2011). In brief, total RNA was extracted from the tissue using TRIzol reagent (Invitrogen, Carlsbad, CA, United States). One microgram of RNA was reverse transcribed to cDNA by using SuperScript III First-Strand Synthesis System (Invitrogen, Carlsbad, CA, United States) and amplified by 40 cycles using primers listed in Supplementary Table 1, and the SYBR Green PCR Master mix reagent (Applied Biosystems, Foster City, CA, United States). 2^–ΔΔCt^ method was applied to determinate relative changes in gene expression levels. Glyceraldehyde 3-phosphate dehydrogenase (GAPDH) mRNA was used to normalize quantitative gene expression data.

### Intestinal Permeability

We quantified intestinal permeability using a FITC-dextran tracer (Sigma, St. Louis, MO, United States). 4 h before blood sample collection, mice were given a 0.5 ml FITC-dextran tracer (4 kDa, 0.4 mg/g body weight) by oral gavage. Mice were then euthanized and blood samples were collected. Samples were left for 30 min to induce coagulation before centrifugation at 6000 rpm for 90 s. FITC-dextran concentration was measured at the excitation wavelength of 488 nm and the emission wavelength of 520 nm (Ma et al., 2018).

### 16S rRNA Gene Sequencing

Fecal samples were randomly selected from each group and collected with a bacterial gDNA Stool Mini Kit (Qiagen, Hilden, Germany). DNA quality assessment was conducted on 1% agarose gel electrophoresis. We used the V3-V4 hypervariable region of 16S rRNA genes as a universal primer (forward 338F: 5′-ACTCCTACGGGAGGCAGCAG-3′; reverse 806R: 5′-GACTACHVGGGTWTCTAAT-3′) to amplify DNA sequences, and generated sequencing libraries using TruSeq DNA PCR-free sample preparation kit. After assessing for quality, the libraries were sequenced on an Illumina Miseq platform with 300 bp paired-end reads (Shanghai Majorbio Bio-pharm Technology, Shanghai, China). The sequencing data were submitted to the National Center of Biotechnology Information Sequence Read Archive Database^1^ with the accession no. PRJNA609459.

The paired-end reads were merged using Fast Length Adjustment of SHort reads (FLASH) software. Poor quality reads were filtered using Trimmomatic software and chimera sequences were removed using the UCHIME algorithm to obtain effective reads. The sequences were aligned into the Silva database of bacterial 16S rRNA genes^2^ at a 70% confidence level using search software. The operational taxonomic units (OTUs) were identified as 1 cluster at the 97% similarity level. Subsequently, the rarefaction curve analysis was presented using Mothur software. Chao1 and Shannon index were used to quantify and compare microbial species richness and alpha diversity. Principal component analysis (PCA) was performed using QIIME^3^ to compare beta diversity. Bacterial taxa within different groups were arranged based on their relative abundance (false discovery rate <0.05). Inner to outer rings were organized according to phylum, class, order, family and genus. All data were analyzed on the free online platform Majorbio Cloud Platform^4^.

### Fecal Microbiota Transfer

Fecal microbiota transfer (FMT) was performed according to the protocol by Chang et al. (Chang et al., 2015) with the following modifications. Briefly, donor mice (*n* = 6) were selected from either HFD group or SYFSF-treated HFD group. After 4 weeks of SYFSF administration, we collected feces from donor mice for 12 weeks consecutively under a laminar flow hood to ensure sterility. 100 mg of fecal material was resuspended into 1 ml of sterile saline and stirred thoroughly. We centrifuged the suspension at 1,200 rpm for 2 min and collected the supernatant for use as transplant material. Fresh transplant material was prepared daily. 8-week-old C57BL/6J male recipient mice (*n* = 6) were fed HFD for 4 weeks and subsequently inoculated with fresh transplant material (100 μl for each mouse) either from the HFD group or SYFSF-treated HFD group by oral gavage for 12 weeks consecutively before euthanization. A schematic diagram for the experimental timeline is shown in Figure 4A. Horizontal fecal transfer from vehicle–treated HFD mice is referred to as HFD receivers. Horizontal fecal transfer from SYFSF-treated mice is referred to as SYFSF receivers.

### Statistical Analysis

Statistical analysis was performed using Prism software (V.8.0; GraphPad Software, La Jolla, CA, United States). Values are presented as means ± standard error (SEM). Comparison of data sets between two groups was performed using a two-tailed unpaired *t*-test. Comparison of data sets between multiple groups was performed using ANOVA. *P* < 0.05 was considered statistically significant.

## Results

### SYFSF Prevented Obesity and Regulated Glucose Metabolism in HFD-Fed Mice

Here, a total of 21 substances were identified from SYFSF by UHPLC-Q-TOF. Calycosin-7-O-glucoside, Ferulic acid, Senkyunolide I, Ononin, Ginsenoside Rd, Ginsenoside Rg1, Formononetin, Ligustilide, Astragaloside II, Levistilide A and other 11 substances were the main constitutes in SYFSF (Supplementary Figure 1). To evaluate the effect of SYFSF on body weight, mice fed with HFD were treated with 11.4 g/kg/d of SYFSF or vehicle by gavage for 16 weeks (Figure 1A). The amount of food intake was standardized throughout in all groups (Figure 1B). HFD-fed mice had a 20.79% higher body weight as compared to the RC-fed group (Figures 1C,D). After the administration of either SYFSF as treatment or vehicle as control, SYFSF-treated HFD-fed mice had a 10.52% lower body weight compared to the HFD-fed mice (Figures 1C,D). GTT revealed glucose intolerance in HFD-fed mice, which were reversed by SYFSF (Figures 1E,F). However, these effects were not seen in RC-fed mice. These data show that the SYFSF administration alleviates HFD-induced obesity and insulin resistance, and the effects are not due to reduced food consumption.

### SYFSF Alleviated Hyperlipidemia and Systemic Inflammation in HFD-Fed Mice

Next, we assessed the effects of SYFSF on lipid metabolism by measuring lipid parameters. In HFD-fed mice, 16-week treatment of SYFSF significantly decreased the concentrations of serum TG, LDL-C and HDL-C as compared to the HFD group (Figures 1G–J). We also measured serum concentrations of TNF-α and IL-1β as obesity is highly associated with systemic inflammation. We found lower serum concentrations of TNF-α and IL-1β in SYFSF-treated HFD-fed mice compared to the HFD-fed mice (Figures 1K,L). Together, these results indicate that SYFSF treatment protects mice from HFD-induced hyperlipidemia and systemic inflammation.

### SYFSF Reduced HFD-Induced Fat Accumulation in Liver and Adipose Tissue

**FIGURE 2:**
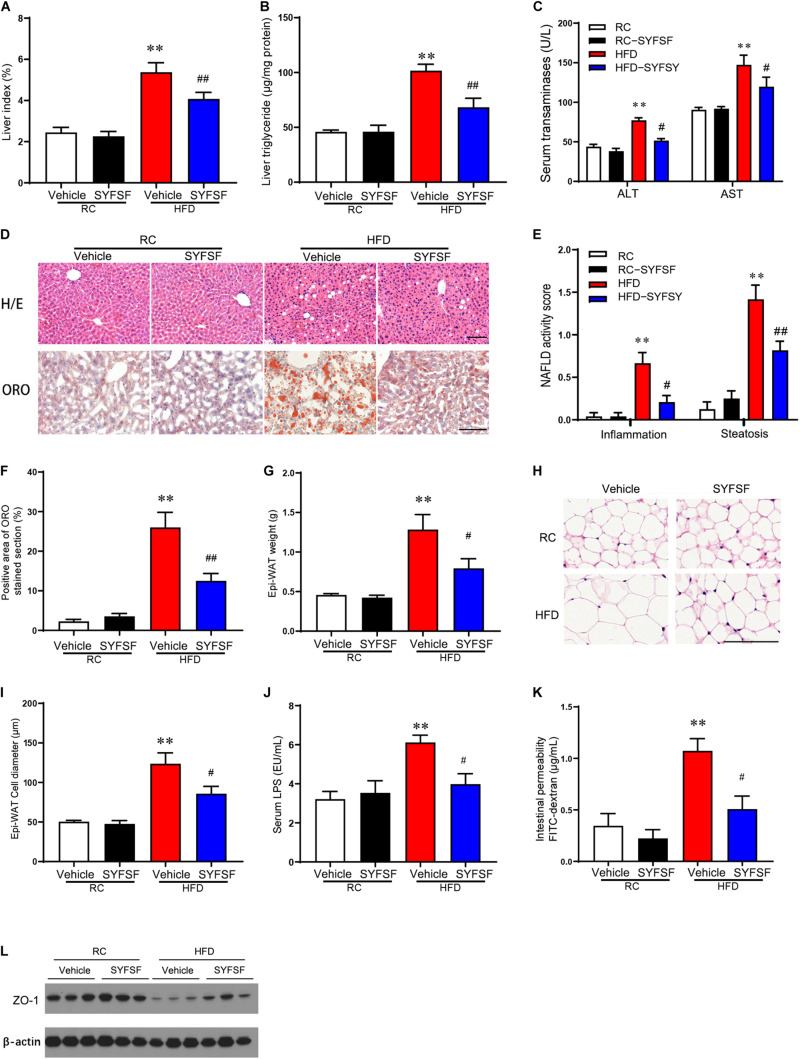
SYFSF prevents fat accumulation in the liver and adipose tissue, and improved gut barrier function. **(A)** Liver index. **(B)** Liver TG. **(C)** Serum ASL and ALT. **(D)** HE staining and Oil Red O staining of sections from the liver. **(E)** NAFLD activity score. **(F)** Oil Red O staining. **(G)** Epi-WAT weight. **(H)** HE staining of sections from Epi-WAT. **(I)** Epi-WAT cell diameter. **(J)** Serum endotoxin. **(K)** Intestinal permeability. **(L)** Expression of ZO-1 in proximal colon. ^∗∗^*P* < 0.01 vs. RC; ^##^*P* < 0.01, ^#^*P* < 0.05 vs. HFD. *n* = 6 per group. Bar = 100 μm.

As shown in Figure 2A, treatment of SYFSF significantly decreased liver index as compared to the HFD group. Liver steatosis and damage induced by HFD were also significantly ameliorated through SYFSF administration, as indicated by decreased hepatic triglyceride content (Figure 2B), and a reduction in plasma AST and ALT concentration (Figure 2C). These results were further supported by H&E and Oil Red O staining (Figures 2D–F). Meanwhile, Epi-WAT weight were markedly reduced in SYFSF-treated HFD-fed mice (Figures 2G–I). Together, these findings indicate that SYFSF treatment can prevent fat accumulation in the liver and adipose tissue of mice fed with HFD.

### SYFSF Improved Gut Barrier Function and Reduced Metabolic Endotoxemia

Previous studies showed that HFD reduced the expression of intestinal tight junction proteins (e.g., ZO-1), hence destroying intestinal barrier integrity and causing transmigration of bacterial LPS into the bloodstream (i.e., metabolic endotoxemia), inflammation and insulin resistance (Cani et al., 2009; Hersoug et al., 2016). We observed that SYFSF treatment significantly reduced serum levels of LPS in HFD-fed mice (Figure 2J). In addition, HFD-fed mice had a decrease in the expression of tight junction protein ZO-1 in the colon and an increase in FITC-dextran levels, which implies increased intestinal permeability and impaired gut barrier function. These observations were significantly attenuated in the SYFSF-treated group (Figures 2K,L). These results show that SYFSF can improve intestinal epithelium integrity and reduce metabolic endotoxemia.

### SYFSF Modified Gut Dysbiosis in HFD-Fed Mice

**FIGURE 3:**
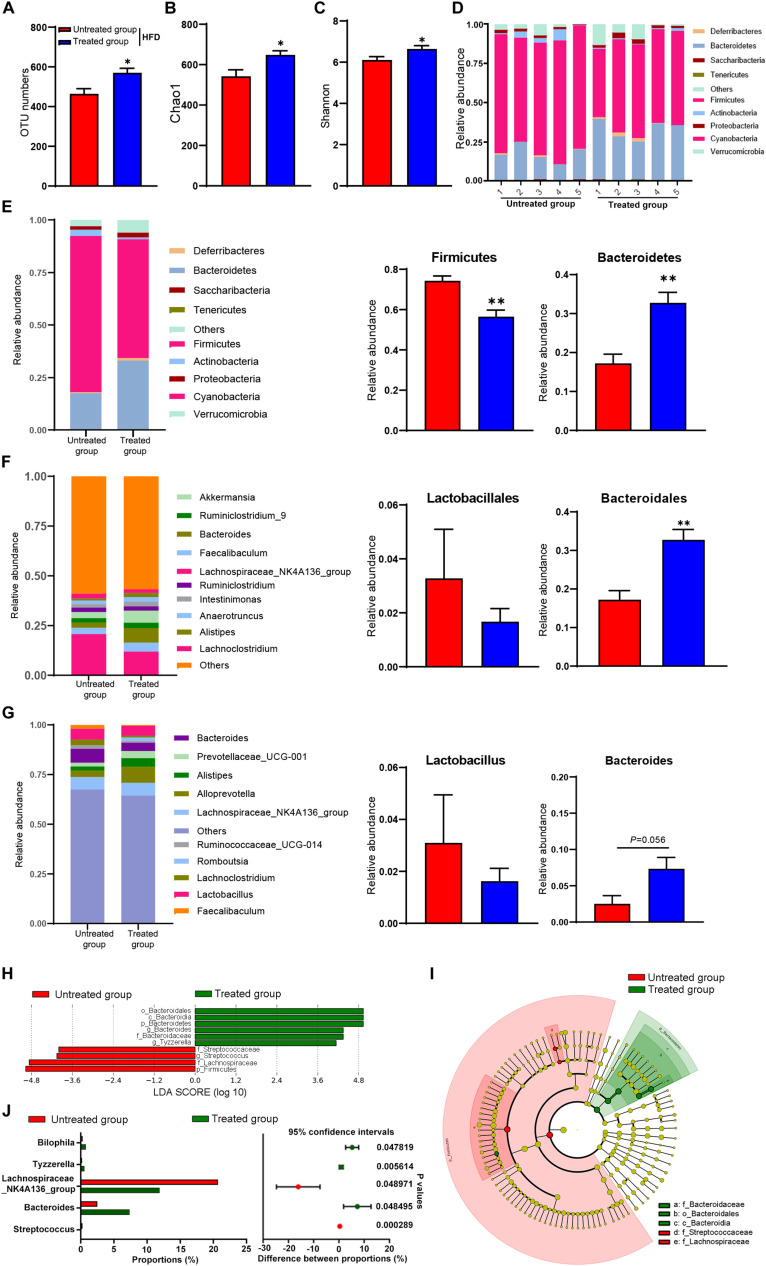
Gut microbiota in response to fecal microbiota transfer (*n* = 5). **(A)** OTU numbers analysis. **(B)** Chao1 index in a-diversity analysis. **(C)** Shannon index in a-diversity analysis. **(D,E)** Microbiota compositions at the phylum level. **(F)** Microbiota compositions at the order level. **(G)** Microbiota compositions at the genus level. **(H)** Biomarker taxons generated from LEfSe analysis. **(I)** Cladogram obtained from LEfSe analysis with presenting various levels (phylum, class, order, family, genus) from inner to outer rings. **(J)** Taxon composition with the top 5 typical bacteria on the genus level (*P* < 0.05, determined by Wilcoxon rank-sum test). Data are presented as means ± SEM. Differences were assessed by ANOVA. ^∗^*P* < 0.05, ^∗∗^*P* < 0.01.

To explore the mechanism of SYFSF’s beneficial effects on obesity, we examined the regulation effect of SYFSF on gut microbiota by sequencing the bacterial 16S rRNA V3–V4 region in mouse feces. An average of 95,592 raw reads was generated from each sample (*n* = 5). Low-quality reads were then filtered, leaving with 92,088 clean tags for analysis and subsequent clustering into OTUs. The number of OTUs in the SYFSF-treated HFD group is significantly higher as compared to that of the HFD group (Figure 3A). Mice in the SYFSF-treated HFD group had significantly greater microbial richness and higher diversity than the HFD group, as evidenced by the higher Chao1 and Shannon indices (Figures 3B,C). Beta diversity of intestinal microbiota was evaluated using uniFrac distance-based PCA. SYFSF-treated HFD-fed mice exhibited a distinctly different composition of microbiota compared to that of HFD-fed mice, while the SYFSF-treated RC-fed mice exhibited similar structure to that of RC-fed mice (Supplementary Figures 2, 3). Overall microbial compositions in different groups were further analyzed. As presented in Figures 3D,E, phylum level analysis showed that HFD feeding resulted in a significant reduction in the relative abundance of Bacteroidetes and a significant increase in that of Firmicutes. Interestingly, there was a significant increase and decrease in abundance of these 2 phyla, respectively after treatment with SYFSF as compared to the untreated group. Further analysis at the order level revealed that HFD-fed mice had a higher abundance of Lactobacillales and a lower abundance of Bacteroidales (Figure 3F). Treatment with SYFSF reversed these alterations (*P* < 0.05 as showed in Bacteroidales expression). Similarly, abundance of Lactobacillus and Bacteroides were decreased and increased, respectively after treatment with SYFSF at the genus level (Figure 3G). Next, we used LEfSe analysis to explore biomarkers presented as taxons in different groups (Figures 3H,I). Compared with the HFD group, SYFSF treatment significantly increased the proportion of Bilophila, Tyzzerella, and Bacteroides and decreased the proportions of Streptococcus and Lachnospiraceae_NK4A136_group (Figure 3J), indicating they would be important targets of SYFSF treatment. Taken together, these data indicate that SYFSF treatment regulates gut microbiota dysbiosis.

### The Gut Microbiota Was Pivotal in Explaining the Therapeutic Effects of SYFSF

**FIGURE 4:**
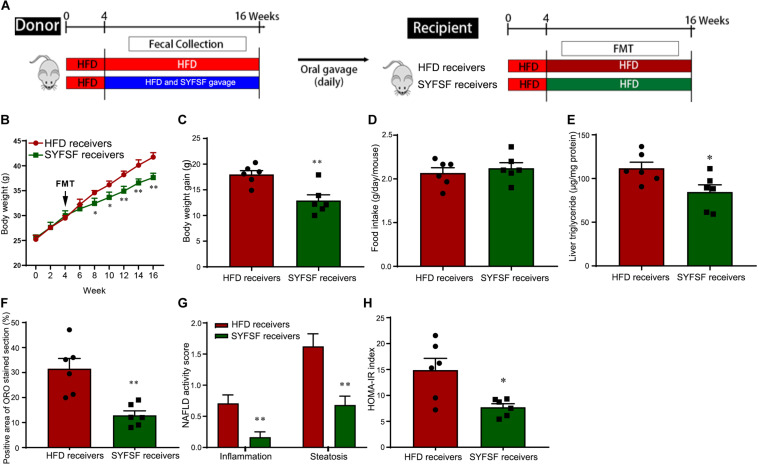
Obesity-associated metabolic syndrome is reversed by feces transfer from SYFSF-treated mice to HFD-fed mice. **(A)** A schematic diagram for the experimental timeline. Horizontal fecal transfer from vehicle–treated HFD mice is referred to as HFD receivers. Horizontal fecal transfer from SYFSF-treated mice is referred to as SYFSF receivers. **(B)** Body weight. **(C)** Body weight gain. **(D)** Food intake. **(E)** Liver TG. **(F)** Liver lipid accumulation was assessed by Oil Red O staining. **(G)** NAFLD activity score. **(H)** HOMA-IR index was calculated according to the formula: fasting insulin (mU/L) × fasting glucose (nmol/L)/22.5. ^∗∗^*P* < 0.01, ^∗^*P* < 0.05 vs. HFD receivers. *n* = 6 per group.

**FIGURE 5:**
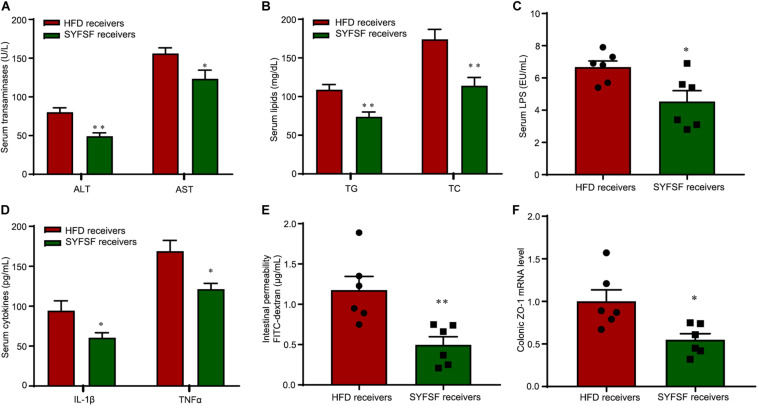
FMT alleviates lipid dysregulation and improves gut barrier function in HFD-fed mice. **(A–D)** Serum ASL, ALT, TG, TC, endotoxin, IL-1β, and TNF-α. **(E)** Intestinal permeability. **(F)** Expression of ZO-1 in proximal colon. ^∗∗^*P* < 0.01, ^∗^*P* < 0.05 vs. HFD receivers. *n* = 6 per group.

There is increasing evidence from animal studies that cross-transfer of intestinal microbiota is an effective means of curbing obesity (Lee et al., 2019). Hence, we performed FMT to determine whether SYFSF exerted its therapeutic effects by modulating gut microbiota. Mice were fed with HFD, and inoculated with fecal transplants either from the SYFSF-treated HFD group (denoted as SYFSF receivers) or from the HFD group (denoted as HFD receivers). Food intake was consistent in both groups (Figure 4D). As shown in Figures 4B,C, SYFSF receivers had a 9.82% decrease in body weight. Intrahepatic TG content in the SYFSF receivers decreased by 24.35% (Figure 4E). Inflammation and steatosis also significantly decreased in the SYFSF receivers as compared to HFD receivers (Figures 4F,G and Supplementary Figure 4). Insulin resistance was also alleviated in SYFSF receivers (Figure 4H). These mice also had improvements in terms of lipid metabolism, liver function, metabolic endotoxemia and inflammation, as evidenced by the decrease in levels of serum ALT, AST, TG, TC, LPS, IL-1β, and TNF-α (Figures 5A–D). In addition, compared with the HFD receivers, SYFSF receivers showed improved permeability (Figures 5E,F). In summary, these data indicate that the beneficial effects of SYFSF on HFD-induced obesity can be replicated through FMT, suggesting the possible role of SYFSF in regulating gut microbiota.

### SYFSF Fecal Microbiota Transfer Modulated the Composition of Intestinal Microbiota in HFD-Fed Mice

**FIGURE 6:**
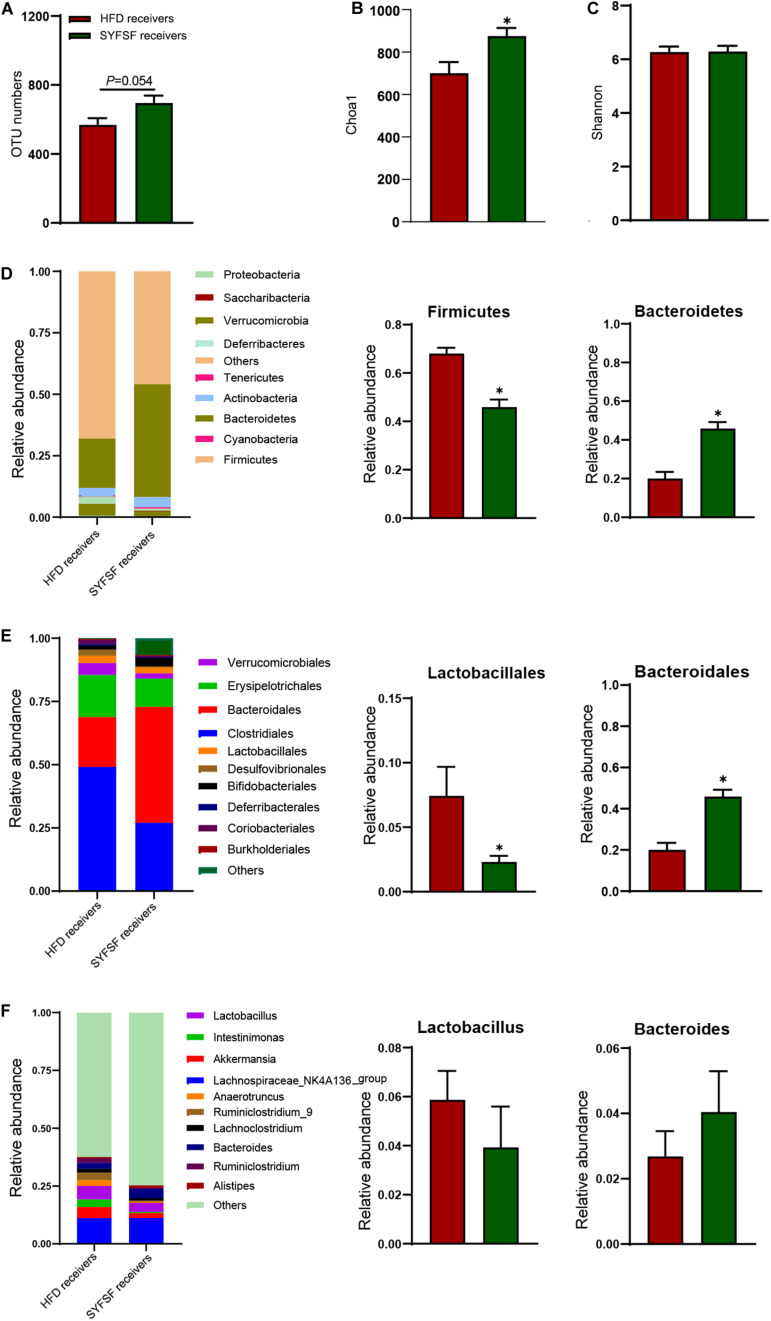
Gut microbiota in response to fecal microbiota transfer (*n* = 6). **(A)** OTU numbers analysis. **(B)** Chao1 index in a-diversity analysis. **(C)** Shannon index in a-diversity analysis. **(D)** Microbiota compositions at the phylum level. **(E)** Microbiota compositions at the order level. **(F)** Microbiota compositions at the genus level. Data are presented as means ± SEM. Differences were assessed by ANOVA. ^∗^*P* < 0.05.

To obtain stronger evidence, we elucidated the gut microbiome profile of FMT recipient mice by sequencing the bacterial 16S rRNA V3 - V4 region. An average of 124,361 raw reads were generated from each sample (*n* = 6). Low-quality reads were filtered, leaving with 120,828 clean tags for analysis and clustering into OTUs. As shown in Figure 6, the overall microbial composition differed at the phylum, order, and genus levels between the SYFSF receivers and HFD receivers. The number of OTUs in SYFSF receivers was higher than that in HFD receivers, but no significant difference was observed. This result is consistent with that of the SYFSF-treated HFD group and HFD group (Figure 6A). SYFSF receivers also exhibited higher species richness, as evidenced by the increased Chao 1 index. However, there was no significant difference in diversity, when we compared the Shannon index (Figures 6B,C). There was a significant difference in the intestinal microbiota composition of both groups upon comparison using the uniFrac distance-based PCA method (Supplementary Figure 5). At the phylum level, the relative abundance of Firmicutes was significantly elevated in the HFD receivers while that of Bacteroidetes was significantly reduced. This phenomenon was reversed in SYFSF receivers (Figure 6D). At the order level, the relative abundance of Lactobacillales was significantly increased in HFD receivers, while that of Bacteroidales significantly decreased. These observations were similarly reversed in SYFSF receivers (Figure 6E). A similar trend was observed for Lactobacillus and Bacteroides at the genus level, but the difference was not statistically significant (Figure 6F). Collectively, these results suggest the efficacy of SYFSF in modulating gut microbiota.

## Discussion

Obesity is emerging as one of the most health-threatening disease in the world (Ford et al., 2010). Hence, there is an urgent need for the development of effective and safe therapeutic agents. Our previous study has shown that, in addition to renal protection, SYFSF was also effective in lowering serum cholesterol, triglyceride and glucose levels (Lv et al., 2017). Leveraging on these results, we speculate that SYFSF may have the effect of reducing obesity.

In our obese mouse model, SYFSF administration significantly decreased body weight. In addition, HFD-fed mice exhibited markedly increased serum levels of TG, TC and glucose intolerance, whereas SYFSF administration exerted a protective effect and counteracted the increased insulin resistance in obese mice. Insulin resistance and abnormalities in lipid metabolism expedite fatty acid synthesis and intrahepatic lipid accumulation, and when accompanied by oxidative stress-mediated lipotoxicity and higher expression of proinflammatory cytokines, cause NAFLD (Takaki et al., 2013; Pisonero-Vaquero et al., 2015; Buzzetti et al., 2016). In this study, liver tissue histopathology of HFD-fed mice revealed significant inflammation and steatosis, while serologic tests revealed elevated levels of ALT and AST. These were ameliorated after SYFSF administration. Collectively, these results imply that SYFSF exhibited robust efficacy against obesity and related metabolic disorders in HFD-fed mice.

Emerging evidence supports the benefits of a favorable profile of gut microbiota in weight management. Many natural products, including plant foods and phytochemicals, have been found to be effective in weight management by modulating gut microbiota (Li et al., 2017; Henning et al., 2018). Since SYFSF is a mixture of many chemical substances, from which we identified 21 substances by UHPLC-Q-TOF analysis. Many of these substances have been reported to affect gut microbiota, including Calycosin-7-O-glucoside (Ruan et al., 2015), Ferulic acid (Ma et al., 2019), Ginsenoside Rd (Han et al., 2020), and Ginsenoside Rg1 (Wang et al., 2020). We believe that the beneficial effects of SYFSF are mostly due to remodeling of gut microbiota. This idea was evidenced in three aspects: (1) Modulating the composition of gut microbiota. By increasing the relative abundance of Firmicutes and decreasing the relative abundance of Bacteroidetes, HFD feeding often causes typical obesity-driven dysbiosis in the intestinal microbiota (Ley et al., 2005, 2006). Our results support this finding to some extent. We found a similar shift in the gut microbiota of HFD-fed mice, and SYFSF administration reversed these alterations in microbial profile from the phylum to the genus level. (2) Improving intestinal barrier function. Intestinal barrier integrity is vital for health and an imbalance in the intestinal barrier integrity can disrupt the balance of microbiota, which leads to various diseases, including intestinal inflammatory disorders and metabolic disorders such as diabetes and obesity (Chelakkot et al., 2018). Our data suggest that HFD initiates metabolic changes that impaired gut barrier function, as evidenced by the decreased expression of ZO-1 in the colon and increased FITC-dextran levels. These damages were significantly reversed after administering SYFSF. (3) Reducing endotoxemia. Metabolic endotoxemia, a condition that is mainly a result of gut microbiota dysbiosis and higher LPS concentration in the blood, often occurs in the HFD-induced chronic low-grade inflammation and related disorders (Lu et al., 2020). Our data demonstrate that SYFSF decreased the HFD-induced elevated levels of endotoxemia in mice.

However, was regulation of gut microbiota a mere concomitant phenomenon, or a potential mechanism through which SYFSF exerted its anti-obesity effects? Recent studies have shown that FMT is a feasible means of confirming the role of gut microbiota in disease pathology (Bibbò et al., 2017; Gupta and Khanna, 2017; Wang et al., 2019), and hence we performed this procedure to validate if SYFSF reduced obesity by altering gut microbiota. We separately transplanted fecal material obtained from HFD-fed and SYFSF-treated HFD-fed donor mice to recipient mice fed with HFD. Results showed that SYFSF receivers had significant improvements in body weight, glucose intolerance, and reductions in serum levels of lipid, liver enzymes and endotoxin, implying that FMT was effective in reducing obesity, regulating glucose and lipid metabolism as well as improving intestinal barrier function and modulating metabolic endotoxemia. In addition, HFD receivers and SYFSF receivers exhibited similar gut microbiota profile (from the phylum to the genus level) to that detected in HFD-fed and SYFSF-treated HFD-fed mice, respectively. This confirms the role of SYFSF in gut microbiota modulation.

Overall, our data show that consumption of SYSFS could attenuate HFD-induced obesity, mouse metabolic disorder parameters, low-grade systemic inflammation, insulin resistance, and fatty liver in mice by altering the gut microbiota. Therefore, SYFSF holds promise for preventing obesity and its related metabolic disorders. Next step metagenomic analysis of SYFSF-treated mouse feces for the identification of the specific bacterial species will provide more valuable information for the exact underlying mechanisms of SYFSF and deserves further investigation.

## Data Availability Statement

The datasets presented in this study can be found in online repositories. The names of the repository/repositories and accession number(s) can be found below: https://www.ncbi.nlm.nih.gov/, PRJNA609459.

## Ethics Statement

The animal study was reviewed and approved by Animal Studies Committee of China-Japan Friendship Hospital.

## Author Contributions

ZW, JL, YGa, and RY performed the experiments, analyzed the data, and wrote the manuscript. JZ edited the manuscript. WS, WC, SA, ZL, and YGu contributed to the discussion. WL, YW, and LP conceived and designed the study. All authors contributed to the article and approved the submitted version.

## Conflict of Interest

The authors declare that the research was conducted in the absence of any commercial or financial relationships that could be construed as a potential conflict of interest.

## Footnotes

1 http://www.ncbi.nlm.nih.gov/sra

2 www.arb-silva-silva.de/

3 http:qiime.org/

4 www.i-sanger.com

## Abbreviations

ALT: alanine aminotransferase
AST: aspartate aminotransferase
FMT: fecal microbiota transfer
GTT: glucose tolerance test
HDL-C: high-density lipoprotein-cholesterol
HFD: high-fat diet
LDL-C: low-density lipoprotein-cholesterol
LPS: lipopolysaccharides
NAFLD: non-alcoholic fatty liver disease
SYFSF: Shen-Yan-Fang-Shuai formula
RC: regular chow
OTU: operational taxonomic unit
PCA: principal component analysis
qPCR: quantitative polymerase chain reaction
TC: total cholesterol
TG: triglycerides
TNF-α: tumor necrosis factor.
