# Supplementary material for: Modulation of the Gut Microbiota by Shen-Yan-Fang-Shuai Formula Improves Obesity Induced by High-Fat Diets
Source: Front Microbiol. 2020 Dec 21;11:564376. doi: 10.3389/fmicb.2020.564376 (PMC7779482; doi:10.3389/fmicb.2020.564376)
Supplement: Supplementary file 1 [file Table_1.DOCX]

Supplementary materials for:

**Modulation of the gut microbiota by Shen-Yan-Fang-Shuai formula improves obesity induced by high-fat diets**

**Supplemental Figures**

**Supplementary Figure 1.** The determination of the related substances in SYFSF by UHPLC-Q-TOF

| DAD chromatograms at 254 nm |
| --- |
| 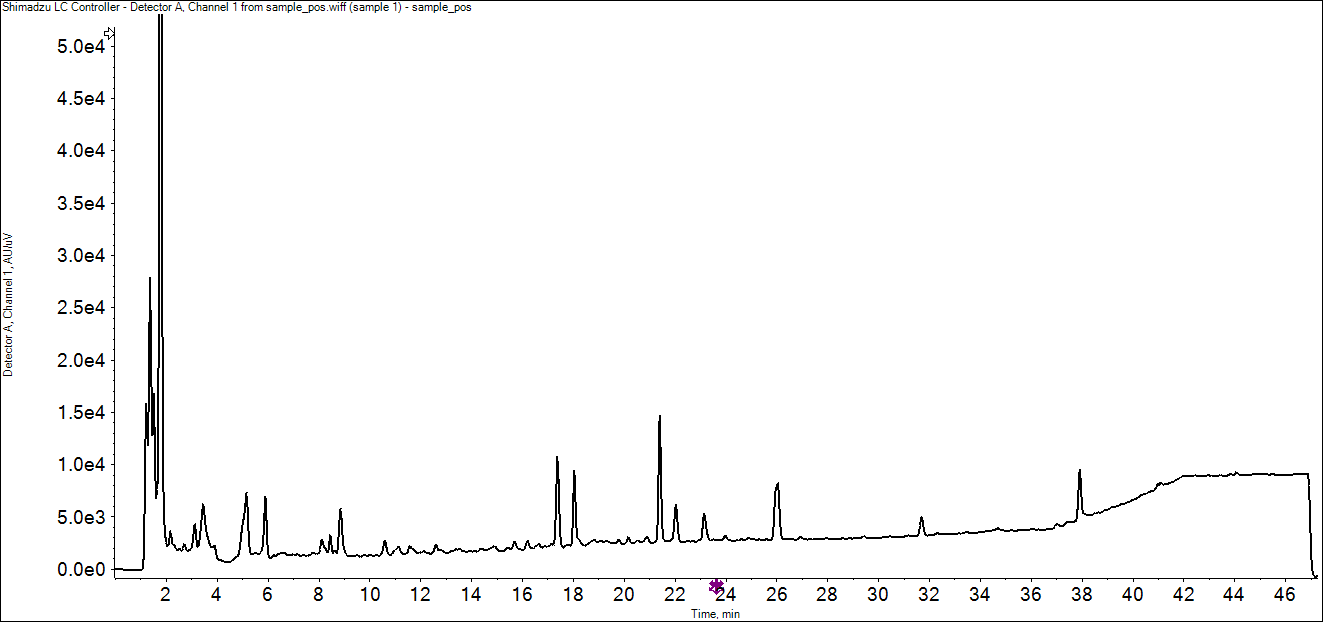  18  15  11, 12  10  9  8  6, 7  5  4  3  1-2 |
| BPC of ESI (+) TOF-MS |
| 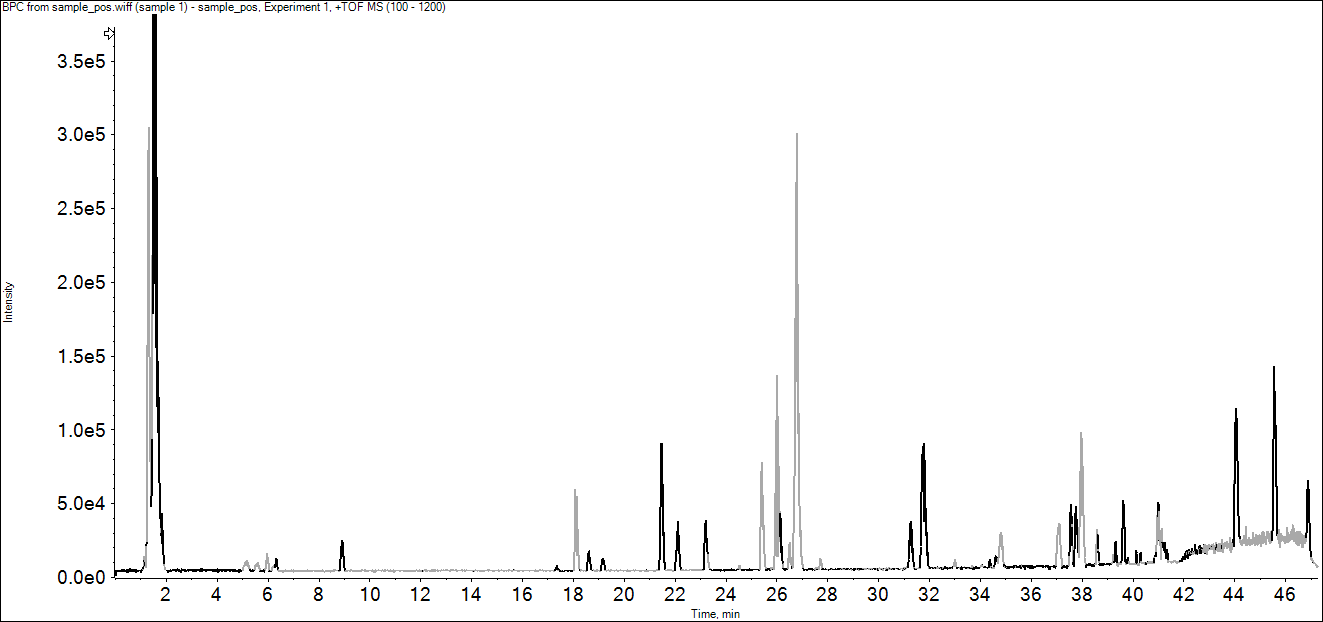  21  20  19  18  16, 17  15  13, 14  11, 12  10  9  8  6, 7  5  4  3  1-2 |

| **Peak** | **Substances** | **Molecular Formula** | **Retention Time** |
| --- | --- | --- | --- |
| 1 | L (+)-Arginine | C6H14N4O2 | 1.29 |
| 2 | Proline | C5H9NO2 | 1.43 |
| 3 | L-tryptophan | C11H12N2O2 | 8.91 |
| 4 | Ferulic acid | C10H10O4 | 17.45 |
| 5 | Calycosin-7-O-glucoside | C22H22O10 | 18.1 |
| 6 | Calycosin-7-O-β-D-glucoside-6''-O-malonate | C25H24O13 | 21.47 |
| 7 | Calycosin | C16H12O5 | 21.5 |
| 8 | Senkyunolide I | C12H16O4 | 22.1 |
| 9 | Ononin | C22H22O9 | 23.19 |
| 10 | (6aR,-11aR)-3-Hydroxy-9,10- dimethoxypterocarpan-3-O-β-D-glucoside | C23H26O10 | 24.5 |
| 11 | Calycosin | C16H12O5 | 26.01 |
| 12 | Formononetin-7-O-β-D-glucoside-6''-O-malonate | C25H24O12 | 26.1 |
| 13 | Ginsenoside Rd | C48H82O18 | 26.71 |
| 14 | Ginsenoside Rg1 | C42H72O14 | 26.8 |
| 15 | Formononetin | C16H12O4 | 31.75 |
| 16 | 20(R)-Ginsenoside Rh1 | C36H62O9 | 34.34 |
| 17 | Senkyunolide A | C12H16O2 | 34.8 |
| 18 | Ligustilide | C12H14O2 | 37.96 |
| 19 | Ginsenoside Rd | C48H82O18 | 39.3 |
| 20 | Astragaloside Ⅱ | C43H70O15 | 39.79 |
| 21 | Levistilide A | C24H28O4 | 40.13 |

**Supplementary Figure 2.** PCA plot analysis from each sample between untreated group and treated group in HFD-fed mice. n = 5 per group.


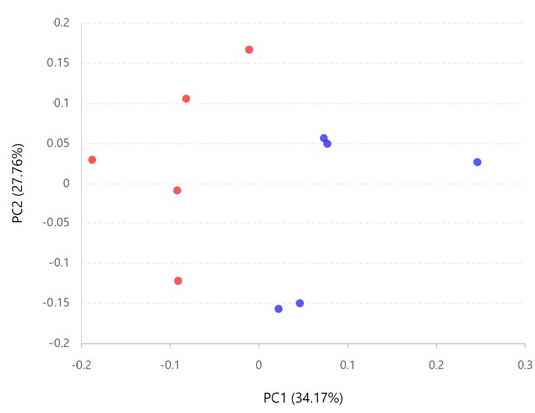


**Supplementary Figure 3.** PCA plot analysis from each sample between untreated group and treated group in RC-fed mice. n = 4 per group.


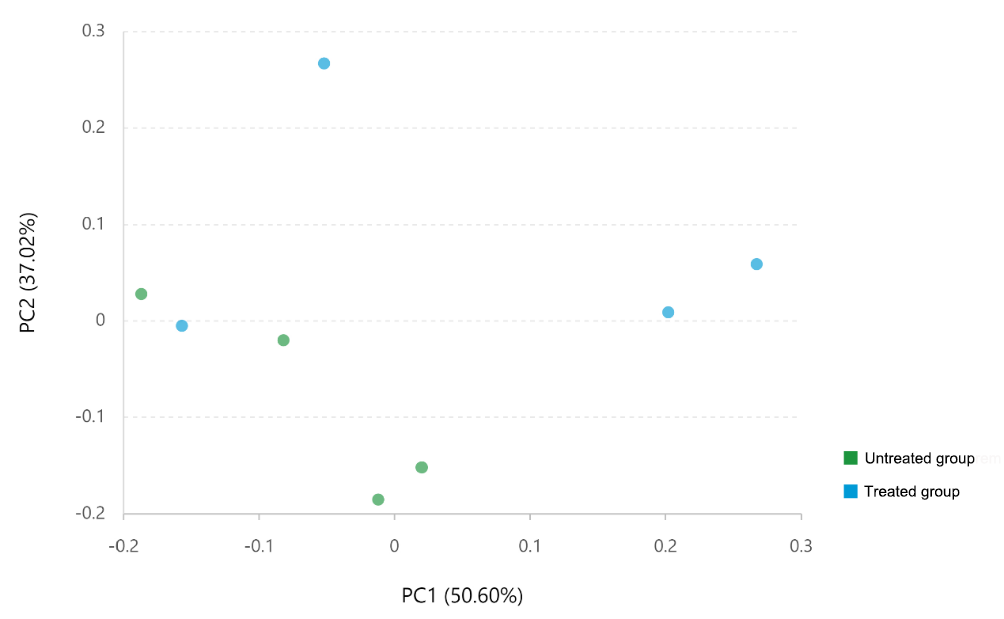


**Supplementary Figure 4.** HE staining and Oil Red O staining of sections from the liver after FMT. Bar= 50 μm


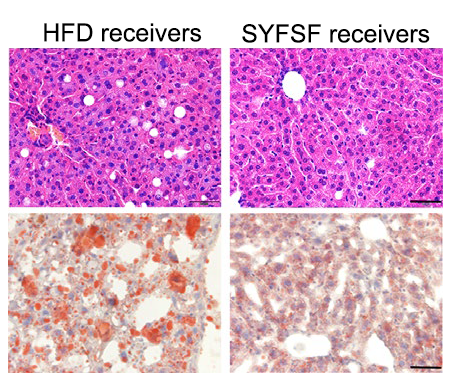


**Supplementary Figure 5.** PCA plot analysis of gut microbiota in response to fecal microbiota transfer.


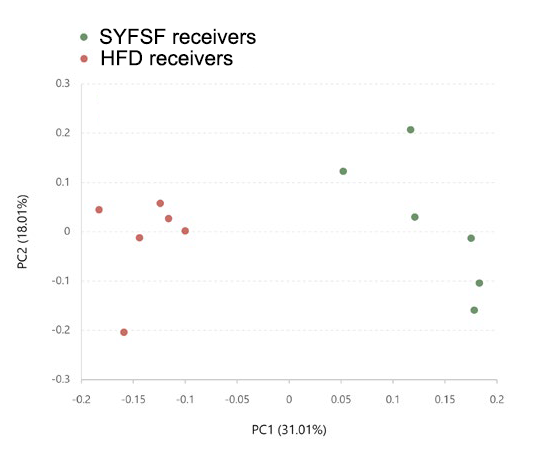


**Supplementary Tables**

**Supplementary Table 1.** Primer Sequences

| Genes | Primer sequence (5′-3′) |
| --- | --- |
| *ZO-1* | F：AGGACACCAAAGCATGTGAG  R: GGCATTCCTGCTGGTTACA |
| *GAPDH* | F: GCATCCACTGGTGCTGCC  R: TCATCATACTTGGCAGGTTTC |

**Supplementary Table 2.** Diversity estimation of the 16S in RC-fed mice. Data are presented as the mean ± SEM (n = 4).

| **Items** | **Untreated group** | **Treated group** | **P-value** |
| --- | --- | --- | --- |
| Chao 1 | 659.48 ± 59.77 | 701.64 ± 21.76 | 0.532 |
| Shannon | 6.39 ± 0.17 | 6.40 ± 0.11 | 0.940 |
| Observed species | 588.55 ± 58.51 | 628.30 ± 31.21 | 0.571 |

**Supplementary Table 3.** Relative abundance of microbial phylum (percentage) in the fecal of RC-fed mice in the untreated group and treated groups. Data are presented as the mean ± SEM (n = 4).

| **Phylum** | **Untreated** | **Treated** | ***P*-value** |
| --- | --- | --- | --- |
| Firmicutes | 51.39 ± 1.12 | 64.13 ± 5.21 | 0.200 |
| Bacteroidetes | 45.87 ± 1.55 | 31.39 ± 4.67 | 0.114 |
| Cyanobacteria | 0.06 ± 0.05 | 0.10 ± 0.06 | 0.686 |
| Tenericutes | 0.11 ± 0.05 | 0.11 ± 0.03 | 0.886 |
| Unidentified phylum | 0.00 ± 0.00 | 0.01 ± 0.00 | 0.243 |
| Deferribacteres | 0.05 ± 0.03 | 0.00 ± 0.00 | 0.110 |
| Proteobacteria | 1.71 ± 0.64 | 2.64 ± 1.14 | 0.486 |
| Saccharibacteria | 0.09 ± 0.04 | 0.15 ± 0.08 | 0.886 |
| Verrucomicrobia | 0.05 ± 0.04 | 0.54 ± 0.34 | 0.114 |
| Actinobacteria | 0.66 ± 0.12 | 0.93 ± 0.46 | 0.886 |

**Supplementary Materials and Methods**

**Extraction and sample preparation**

Aliquots of 0.020 g of lyophilized, pulverized and homogenized powder were extracted with 5mL methanol/water (v/v = 1:1) in the sonicator for 20 min at room temperature. The extract was filtered through a 0.22 μm membrane. A 3 μL aliquot of extract was injected into the analytical column for analysis.

**UHPLC-DAD-Q-TOF conditions**

As we know that the chemical components of traditional Chinese medicine are complex, UHPLC-Q-TOF instrument is often applied for the detection of chemical composition of traditional Chinese medicine. UHPLC combined with small particle chromatographic columns can separate different compounds well. Due to the high resolution and fast scanning speed, the chemical components in Chinese medicine can be determined comprehensively and accurately by the Q-TOF instrument. Since most of the chemical components in Chinese medicine are polar and medium-polar small molecule compounds, the electrospray ion source (ESI) is selected as the ionization method.

A Shimadzu UHPLC system (Kyoto, Japan) equipped a LC-30AD solvent delivery system, a SIL-30AC autosampler, a CTO-30A column oven, a DGU-20A3 degasser and a CBM-20A controller. The separation of the compounds was carried out on a Waters ACQUITY UPLC HSS T3 (2.1X100 mm, 1.8 um) operated at 35°C. The mobile phase, which consists of 0.1% formic acid in water (A) and acetonitrile (B) as mobile phase, was delivered at a flow rate of 0.4 mL/min under a gradient program. The gradient system was 0–1 min, 2% B; 1–35 min, 2–60% B; 35–40 min, 60–95% B; 40–45 min, 95 %B; 45.0-45.1 min, 90-5% B; 45.1-50 min, 2% B. The diode-array detector was set to monitor at 254 nm, and the online UV spectra were recorded in the scanning range of 190–400 nm.

The mass spectra were acquired using a TripleTOF™ 5600+ system with a Duo Spray source (SCIEX, Foster City, CA, USA) in negative and positive ESI mode. Optimized parameter for negative and positive mode was as follows: the ion spray voltage was set to 5,500 (positive ion mode) and −4,500 V (negative ion mode); the Turbo V spray temperature, 550°C; nebulizer gas (Gas 1), 50 psi; heater gas (Gas 2), 60 psi; collision gas, medium; the curtain gas was kept at 30 psi; and declustering potential, 60 (positive ion mode) and −60 V (negative ion mode). The collision energy was set at 35 (positive ion mode) and −35 V (negative ion mode), and the collision energy spread was 15 V for MS/MS experiments. The data was acquired with IDA (information dependent acquisition) method. For TOF-MS and TOF-MS/MS analysis, the spectra covered the range from m/z 100 to 1,200 Da and 50-1200 Da. The data were analyzed by Peak View Software™ 2.2 (SCIEX, Foster City, CA, USA).

For data collection, it is necessary to obtain unknown TOF-MS and TOF- MS/MS accurate mass information for identifying the unknown compounds in SYFSF. The method adopts IDA (information dependent acquisition) acquisition method (see the figure below). This acquisition method acquires TOF-MS and TOF-MS/MS information at the same time in a single injection.


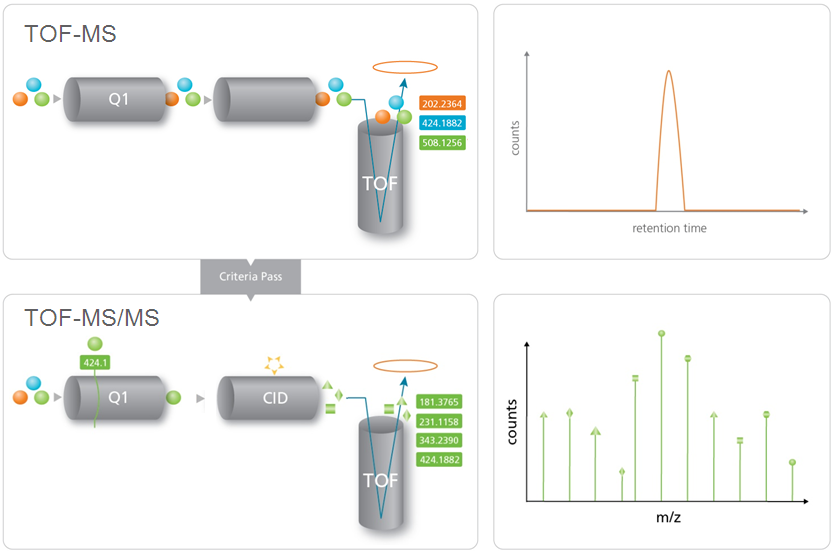


The compounds identification is based on several aspects: 1. Retention time (if there is a standard substance) < 0.2 min 2. The accuracy of the TOF-MS < 5 ppm 3. The database search result score > 70 points, the higher the score, the more confidence. The figure below is the identification result of Calycosin, the retention time is 26.01min, the mass accuracy is -0.8 ppm, and the library search result score is 95.1. From the above results, it can be identified as Calycosin. If the chemical substance does not exist in the database, it can be analyzed by consulting related literature or related databases.
